# A Coding Theorem for f-Separable Distortion Measures

**DOI:** 10.3390/e20020111

**Published:** 2018-02-08

**Authors:** Yanina Shkel, Sergio Verdú

**Affiliations:** Department of Electrical Engineering, Princeton University, Princeton, NJ 08544, USA

**Keywords:** rate-distortion function, f-separable distortion measure, subadditive distortion measure

## Abstract

In this work we relax the usual separability assumption made in rate-distortion literature and propose f-separable distortion measures, which are well suited to model non-linear penalties. The main insight behind f-separable distortion measures is to define an *n*-letter distortion measure to be an f-mean of single-letter distortions. We prove a rate-distortion coding theorem for stationary ergodic sources with f-separable distortion measures, and provide some illustrative examples of the resulting rate-distortion functions. Finally, we discuss connections between f-separable distortion measures, and the subadditive distortion measure previously proposed in literature.

## 1. Introduction

Rate-distortion theory, a branch of information theory that studies models for lossy data compression, was introduced by Claude Shannon in [1]. The approach of [1] is to model the information source with distribution $P_{X}$ on X, a reconstruction alphabet $\hat{X}$, and a distortion measure $d:X\times \hat{X}\to \left[0,\infty \right)$. When the information source produces a sequence of *n* realizations, the source $P_{X^{n}}$ is defined on $X^{n}$ with reconstruction alphabet ${\hat{X}}^{n}$, where $X^{n}$ and ${\hat{X}}^{n}$ are *n*-fold Cartesian products of X and $\hat{X}$. In that case, [1] extended the notion of a single-letter distortion measure to the *n*-letter distortion measure, $d^{n}:X^{n}\times {\hat{X}}^{n}\to \left[0,\infty \right)$, by taking an arithmetic average of single-letter distortions,

(1) $$\begin{array}{c} d^{n}\left(x^{n},{\hat{x}}^{n}\right)=\frac{1}{n}\sum_{i=1}^{n}d\left(x_{i},{\hat{x}}_{i}\right). \end{array}$$

Distortion measures that satisfy (1) are referred to as *separable* (also additive, per-letter, averaging); the separability assumption has been ubiquitous throughout rate-distortion literature ever since its inception in [1].

On the one hand, the separability assumption is quite natural and allows for a tractable characterization of the fundamental trade-off between the rate of compression and the average distortion. For example, in the case when $X^{n}$ is a stationary and memoryless source the rate-distortion function, which captures this trade-off, admits a simple characterization:

(2) $$\begin{array}{c} R\left(d\right)=\underset{P_{\hat{X}|X}:E\left[d(X,\hat{X})\right]\leq d}{\inf}I\left(X;\hat{X}\right). \end{array}$$

On the other hand, the separability assumption is very restrictive as it only models distortion penalties that are *linear* functions of the per-letter distortions in the source reproduction. Real-world distortion measures, however, may be highly *non-linear*; it is desirable to have a theory that also accommodates non-linear distortion measures. To this end, we propose the following definition:

**Definition** **1** (f-separable distortion measure). *Let f(z) be a continuous, increasing function on [0,∞). An n-letter distortion measure $d^{n}\left(\cdot ,\cdot \right)$ is f-separable with respect to a single-letter distortion d(·,·) if it can be written as*

(3) $$\begin{array}{c} d^{n}\left(x^{n},{\hat{x}}^{n}\right)=f^{-1}\left(\frac{1}{n}\sum_{i=1}^{n}f\left(d(x_{i},{\hat{x}}_{i})\right)\right). \end{array}$$

**Figure 1:**
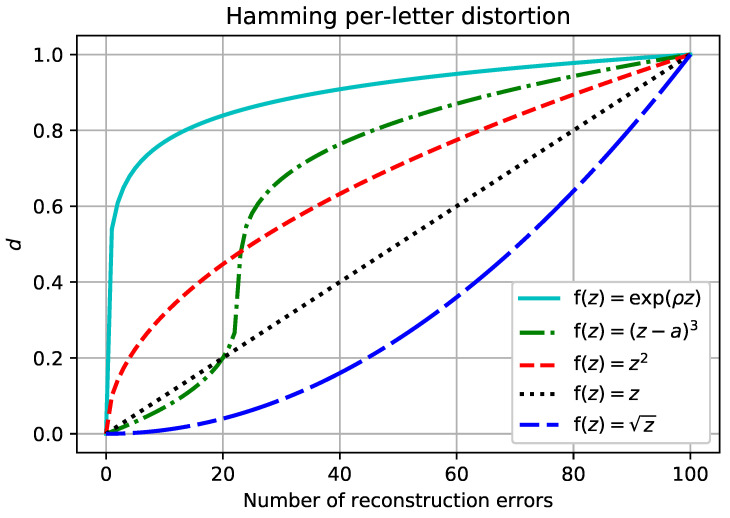
The number of reconstruction errors for an information source with 100 bits vs. the penalty assessed by f-separable distortion measures based on the Hamming single-letter distortion. The f(z)=z plot corresponds to the separable distortion. The f-separable assumption accommodates all of the other plots, and many more, with the appropriate choice of the function f.

For f(z)=z this is the classical separable distortion set up. By selecting f appropriately, it is possible to model a large class of non-linear distortion measures, see Figure 1 for illustrative examples.

In this work, we characterize the rate-distortion function for stationary and ergodic information sources with f-separable distortion measures. In the special case of memoryless and stationary sources we obtain the following intuitive result:

(4) $$\begin{array}{c} R_{f}\left(d\right)=\underset{P_{\hat{X}|X}:E\left[f\left(d(X,\hat{X})\right)\right]\leq f\left(d\right)}{\inf}I\left(X;\hat{X}\right). \end{array}$$

A pleasing implication of this result is that much of rate-distortion theory (e.g., the Blahut-Arimoto algorithm) developed since [1] can be leveraged to work under the far more general f-separable assumption.

The rest of this paper is structured as follows. The remainder of Section 1 overviews related work: Section 1.1 provides the intuition behind Definition 1, Section 1.2 reviews related work in other compression problems, and Section 1.3 connects f-separable distortion measures with sub-additive distortion measures. Section 2 formally sets up the problem and demonstrates why convexity of the rate-distortion function does not always hold under the f-separable assumption. Section 3 presents our main result, Theorem 1, as well as some illustrative examples. Additional discussion about problem formulation and sub-additive distortion measures is given in Section 4. We conclude the paper in Section 5.

### 1.1. Generalized f-Mean and Rényi Entropy

To understand the intuition behind Definition 1, consider aggregating *n* numbers $\left(z_{1},\cdots ,z_{n}\right)$ by defining a sequence of functions (indexed by *n*)

(5) $$\begin{array}{c} M_{n}\left(z\right)=f^{-1}\left(\frac{1}{n}\sum_{i=1}^{n}f\left(z_{i}\right)\right) \end{array}$$

where f is a continuous, increasing function on $\left[z_{\min},z_{\max}\right]$, $z_{\min}=\min{\left\{z_{i}\right\}}_{i=1}^{n}$, and $z_{\max}=\max{\left\{z_{i}\right\}}_{i=1}^{n}$. It is easy to see that (5) satisfies the following properties:

1. $M_{n}\left(z\right)$ is continuous and monotonically increasing in each $z_{i}$,
2. $M_{n}\left(z\right)$ is a symmetric function of each $z_{i}$,
3. If $z_{i}=z$ for all *i*, then $M_{n}\left(z\right)=z$,
4. For any m≤n
  (6) $$\begin{array}{c} M_{n}\left(z\right)=M_{n}\left(M_{m}\left(z_{1}^{m}\right),\cdots ,M_{m}\left(z_{1}^{m}\right),z_{m+1},\cdots ,z_{n}\right). \end{array}$$

Moreover, it is shown in [2] that any sequence of functions $M_{n}$ that satisfies these properties must have the form of Equation (5) for some continuous, increasing f. The function $M_{n}$ is referred to as “Kolmogorov mean”, “quasi-arithmetic mean”, or “generalized f-mean”. The most prominent examples are the geometric mean, f(z)=logz, and the root-mean-square, $f\left(z\right)=z^{2}$.

The main insight behind Definition 1 is to define an *n*-letter distortion measure to be an f-mean of single-letter distortions. The f-separable distortion measures include all *n*-letter distortion measures that satisfy the above properties, with the last property saying that the non-linear “shape” of distortion measure (cf. Figure 1) is independent of *n*.

Finally, we note that Rényi also arrived at his well-known family of entropies [3] by taking an f-mean of the information random variable:

(7) $$\begin{array}{cc} H_{\alpha }\left(X\right) & =f_{\alpha }^{-1}E\left[f_{\alpha }\left({ı}_{X}\left(X\right)\right)\right],\;\alpha \in \left(0,1\right)\cup \left(1,\infty \right) \end{array}$$

where the information at *x* is

(8) $$\begin{array}{cc} {ı}_{X}\left(x\right) & =\log\frac{1}{P_{X}\left(x\right)}. \end{array}$$

Rényi [3] limited his consideration to functions of the form $f_{\alpha }\left(z\right)=\exp\left\{(1-\alpha )z\right\}$ in order to ensure that entropy is additive for independent random variables.

### 1.2. Compression with Non-Linear Cost

Source coding with non-linear cost has already been explored in the variable-length lossless compression setting. Let ℓ(x) denote the length of the encoding of *x* by a given variable length code. Campbell [4,5] proposed minimizing a cost function of the form

(9) $$\begin{array}{c} f^{-1}E\left[f(\ell (X))\right], \end{array}$$

instead of the usual expected length. The main result of [4,5] is that for

(10) $$\begin{array}{c} f_{t}\left(z\right)=\exp\left\{tz\right\},\;t\in \left(-1,0\right)\cup \left(0,\infty \right), \end{array}$$

the fundamental limit of such setup is Rényi entropy of order $\alpha =\frac{1}{t+1}$. For more general f, this problem was handled by Kieffer [6], who showed that (9) has a fundamental limit for a large class of functions f. That limit is Rényi entropy of order $\alpha =\frac{1}{t+1}$ with

(11) $$\begin{array}{c} t=\lim_{z\to \infty }\frac{f^{\prime \prime }\left(z\right)}{f^{\prime }\left(z\right)}. \end{array}$$

More recently, a number of works [7,8,9] studied related source coding paradigms, such as guessing and task encoding. These works also focused on the exponential functions given in (10); in [7,8] Rényi entropy is shown to be a fundamental limit yet again.

### 1.3. Sub-Additive Distortion Measures

A notable departure from the separability assumption in rate-distortion theory is sub-additive distortion measures discussed in [10]. Namely, a distortion measure is sub-additive if

(12) $$\begin{array}{c} d^{n}\left(x^{n},{\hat{x}}^{n}\right)\leq \frac{1}{n}\sum_{i=1}^{n}d\left(x_{i},{\hat{x}}_{i}\right). \end{array}$$

In the present setting, an f-separable distortion measure is sub-additive if f is concave:

(13) $$\begin{array}{c} d^{n}\left(x^{n},{\hat{x}}^{n}\right)=f^{-1}\left(\frac{1}{n}\sum_{i=1}^{n}f\left(d(x_{i},{\hat{x}}_{i})\right)\right)\leq \frac{1}{n}\sum_{i=1}^{n}d\left(x_{i},{\hat{x}}_{i}\right). \end{array}$$

Thus, the results for sub-additive distortion measures, such as the convexity of the rate-distortion function, are applicable to f-separable distortion measures when f is concave.

## 2. Preliminaries

Let *X* be a random variable defined on X with distribution $P_{X}$, with reconstruction alphabet $\hat{X}$, and a distortion measure $d:X\times \hat{X}\to \left[0,\infty \right)$. Let M={1,⋯,M} be the message set.

**Definition** **2** (Lossy source code). *A lossy source code (g,c) is a pair of mappings,*

(14) $$\begin{array}{cc} g & :X\to M \end{array}$$

(15) $$\begin{array}{cc} c & :M\to \hat{X}. \end{array}$$

A lossy source-code (g,c) is an (M,d)-lossy source code on $\left(X,\hat{X},d\right)$ if

(16) $$\begin{array}{c} E\left[d(X,c(g(X))\right]\leq d. \end{array}$$

A lossy source code (g,c) is an (M,d,ϵ)-lossy source code on $\left(X,\hat{X},d\right)$ if

(17) $$\begin{array}{c} P\left[d(X,c(g(X))>d\right]\leq \epsilon . \end{array}$$

**Definition** **3.** *An information source X is a stochastic process*

(18) $$\begin{array}{c} X={\left\{X^{n}=\left(X_{1},\cdots ,X_{n}\right)\right\}}_{n=1}^{\infty }. \end{array}$$

If (g,c) is an (M,d)-lossy source code for $X^{n}$ on $\left(X^{n},{\hat{X}}^{n},d^{n}\right)$, we say (g,c) is an (n,M,d)-lossy source code. Likewise, an (M,d,ϵ)-lossy source code for $X^{n}$ on $\left(X^{n},{\hat{X}}^{n},d^{n}\right)$ is an (n,M,d,ϵ)-lossy source code.

### 2.1. Rate-Distortion Function (Average Distortion)

**Definition** **4.** *Let a sequence of distortion measures $\left\{d^{n}\right\}$ be given. The rate-distortion pair (R,d) is achievable if there exists a sequence of $\left(n,M_{n},d_{n}\right)$-lossy source codes such that*

$$\begin{array}{c} \underset{n\to \infty }{\lim\sup}\frac{1}{n}\log M_{n}\leq R,\;and\;\underset{n\to \infty }{\lim\sup}\;d_{n}\leq d. \end{array}$$

Our main object of study is the following rate-distortion function with respect to f-separable distortion measures.

**Definition** **5.** *Let $\left\{d^{n}\right\}$ be a sequence of f-separable distortion measures. Then,*

(19) $$\begin{array}{c} R_{f}\left(d\right)=\inf\left\{R:\left(R,d\right)is\;achievable\right\}. \end{array}$$

If f is the identity, then we omit the subscript f and simply write R(d).

### 2.2. Rate-Distortion Function (Excess Distortion)

It is useful to consider the rate-distortion function for f-separable distortion measures under the excess distortion paradigm.

**Definition** **6.** *Let a sequence of distortion measures $\left\{d^{n}\right\}$ be given. The rate-distortion pair (R,d) is (excess distortion) achievable if for any γ>0 there exists a sequence of $\left(n,M_{n},d+\gamma ,\epsilon_{n}\right)$-lossy source codes such that*

$$\begin{array}{c} \underset{n\to \infty }{\lim\sup}\frac{1}{n}\log M_{n}\leq R,\;and\;\underset{n\to \infty }{\lim\sup}\;\epsilon_{n}=0. \end{array}$$

**Definition** **7.** *Let $\left\{d^{n}\right\}$ be a sequence of f-separable distortion measures. Then,*

(20) $$\begin{array}{cc} R_{f}^{\prime }\left(d\right)=\inf\{R & :(R,d)\;is\;(excess\;distortion)\;achievable\}. \end{array}$$

Characterizing the f-separable rate-distortion function is particularly simple under the excess distortion paradigm, as shown in the following lemma.

**Lemma** **1.** *Let the single-letter distortion d and an increasing, continuous function f be given. Then,*

(21) $$\begin{array}{c} R_{f}^{\prime }\left(d\right)={\tilde{R}}^{\prime }\left(f\left(d\right)\right) \end{array}$$

*where ${\tilde{R}}^{\prime }\left(d\right)$ is computed with respect to $\tilde{d}\left(x,\hat{x}\right)=f\left(d\left(x,\hat{x}\right)\right)$.*

**Proof.**  Let $\left\{d^{n}\right\}$ be a sequence of f-separable distortions based on d(·,·) and let $\left\{{\tilde{d}}^{n}\right\}$ be a sequence of separable distortion measures based on $\tilde{d}\left(\cdot ,\cdot \right)=f\left(d\left(\cdot ,\cdot \right)\right)$.

Since f is increasing and continuous at *d*, then for any γ>0 there exists $0<\tilde{\gamma }$ such that

(22) $$\begin{array}{c} f\left(d+\gamma \right)-f\left(d\right)=\tilde{\gamma }. \end{array}$$

The reverse is also true by continuity of f: for any $\tilde{\gamma }>0$ there exists γ>0 such that (22) is satisfied.

Any source code $\left(g_{n},c_{n}\right)$ is an $\left(n,M_{n},d+\gamma ,\epsilon_{n}\right)$-lossless code under f-separable distortion $d^{n}$ if and only if $\left(g_{n},c_{n}\right)$ is also an $\left(n,M_{n},f\left(d\right)+\tilde{\gamma },\epsilon_{n}\right)$-lossless code under separable distortion ${\tilde{d}}^{n}$. Indeed,

(23) $$\begin{array}{cc} \epsilon_{n} & \geq P\left[d^{n}\left(X^{n},c_{n}\left(g_{n}\left(X^{n}\right)\right)\right)\geq d+\gamma \right] \end{array}$$

(24) $$\begin{array}{c} =P\left[f^{-1}\left(\frac{1}{n}\sum_{i=1}^{n}f\left(d\left(X_{i},{\hat{X}}_{i}\right)\right)\right)\geq d+\gamma \right] \end{array}$$

(25) $$\begin{array}{c} =P\left[\frac{1}{n}\sum_{i=1}^{n}f\left(d\left(X_{i},{\hat{X}}_{i}\right)\right)\geq f\left(d+\gamma \right)\right] \end{array}$$

(26) $$\begin{array}{c} =P\left[{\tilde{d}}^{n}\left(X^{n},c_{n}\left(g_{n}\left(X^{n}\right)\right)\right)\geq f\left(d\right)+\tilde{\gamma }\right] \end{array}$$

where ${\hat{X}}^{n}=c_{n}\left(g_{n}\left(X^{n}\right)\right)$. It follows that (R,d) is (excess distortion) achievable with respect to $\left\{d^{n}\right\}$ if and only if (R,f(d)) is (excess distortion) achievable with respect to $\left\{{\tilde{d}}^{n}\right\}$. The lemma statement follows from this observation and Definition 6. ☐

### 2.3. f-Separable Rate-Distortion Functions and Convexity

While it is a well-established result in rate-distortion theory that all separable rate-distortion functions are convex ([11], Lemma 10.4.1), this need not hold for f-separable rate-distortion functions.

The convexity argument for separable distortion measures is based on the idea of time sharing; that is, suppose there exists an $\left(n_{1},M_{1},d_{1}\right)$-lossy source code of blocklength $n_{1}$ and an $\left(n_{2},M_{2},d_{2}\right)$-lossy source code of blocklength $n_{2}$. Then, there exists an (n,M,d)-lossy source code of blocklength *n* with $M=M_{1}M_{2}$ and $d=\frac{n_{1}}{n_{1}+n_{2}}d_{1}+\frac{n_{1}}{n_{1}+n_{2}}d_{2}$: such a code is just a concatenation of codes over blocklengths $n_{1}$ and $n_{2}$. The distortion *d* is achievable since

(27) $$\begin{array}{c} d^{n_{1}}\left(x^{n_{1}},{\hat{x}}^{n_{1}}\right)=\frac{1}{n_{1}}\sum_{i=1}^{n_{1}}d\left(x_{i},{\hat{x}}_{i}\right)=d_{1} \end{array}$$

and letting $n=n_{1}+n_{2}$,

(28) $$\begin{array}{c} d^{n_{2}}\left(x_{n_{1}+1}^{n},{\hat{x}}_{n_{1}+1}^{n}\right)=\frac{1}{n_{2}}\sum_{i=n_{1}+1}^{n}d\left(x_{i},{\hat{x}}_{i}\right)=d_{2}. \end{array}$$

Time sharing between the two schemes gives

(29) $$\begin{array}{c} d^{n}\left(x^{n},{\hat{x}}^{n}\right)=\frac{1}{n}\sum_{i=1}^{n}d\left(x_{i},{\hat{x}}_{i}\right)=\frac{n_{1}}{n}d_{1}+\frac{n_{2}}{n}d_{2}. \end{array}$$

However, this bound on the distortion need not hold for f-separable distortions. Consider f which is strictly convex and suppose

(30) $$\begin{array}{c} f^{-1}\left(\frac{1}{n_{1}}\sum_{i=1}^{n_{1}}f\left(d(x_{i},{\hat{x}}_{i})\right)\right)=d_{1},\;f^{-1}\left(\frac{1}{n_{2}}\sum_{i=n_{1}+1}^{n}f\left(d(x_{i},{\hat{x}}_{i})\right)\right)=d_{2}. \end{array}$$

We can write

(31) $$\begin{array}{c} f^{-1}\left(\frac{1}{n}\sum_{i=1}^{n}f\left(d(x_{i},{\hat{x}}_{i})\right)\right)=f^{-1}\left(\frac{n_{1}}{n}f\left(d_{1}\right)+\frac{n_{2}}{n}f\left(d_{2}\right)\right)>\frac{n_{1}}{n}d_{1}+\frac{n_{2}}{n}d_{2}. \end{array}$$

Thus, concatinating the two schemes together does not guarantee that the distortion assigned by the f-separable distortion measure is bounded by *d*.

## 3. Main Result

In this section we make the following standard assumptions, see [12].

1. X is a stationary and ergodic source.
2. The single-letter distortion function d(·,·) and the continuous and increasing function f(·) are such that
  (32) $$\begin{array}{c} \underset{\hat{x}\in \hat{X}}{\inf}E\left[f(d\left(X,\hat{x}\right))\right]<\infty . \end{array}$$
3. For each d>0, there exists a countable subset $\left\{{\hat{x}}_{i}\right\}$ of $\hat{X}$ and a countable measurable partition $\left\{E_{i}\right\}$ of X such that $d(x,{\hat{x}}_{i})\leq d$, $x\in E_{i}$ for each ${\hat{x}}_{i}$, and
  (33) $$\begin{array}{c} \sum_{i}P_{X_{1}}\left(E_{i}\right)\log\frac{1}{P_{X_{1}}\left(E_{i}\right)}<\infty . \end{array}$$

**Theorem** **1.** *Under the stated assumptions, the rate-distortion function is given by*

(34) $$\begin{array}{c} R_{f}\left(d\right)=\tilde{R}\left(f\left(d\right)\right) \end{array}$$

*where*

(35) $$\begin{array}{c} \tilde{R}\left(f\left(d\right)\right)=\lim_{n\to \infty }\underset{P_{{\hat{X}}^{n}|X^{n}}:\frac{1}{n}\sum_{i=1}^{n}E\tilde{d}\left(X_{i},{\hat{X}}_{i}\right)\leq f\left(d\right)}{\inf}\frac{1}{n}I\left(X^{n};{\hat{X}}^{n}\right) \end{array}$$

*is the rate-distortion function computed with respect to the separable distortion measure given by $\tilde{d}\left(x,\hat{x}\right)=f\left(d\left(x,\hat{x}\right)\right)$.*

*For stationary memoryless sources (34) particularizes to*

(36) $$\begin{array}{c} R_{f}\left(d\right)=\underset{P_{\hat{X}|X}:E\left[f\left(d(X,\hat{X})\right)\right]\leq f\left(d\right)}{\inf}I\left(X;\hat{X}\right). \end{array}$$

**Proof.**  Equations (35) and (36) are widely known in literature (see, for example, [10,11,13]); it remains to show (34). Under the stated assumptions,

(37) $$\begin{array}{c} R_{f}\left(d\right)\overset{\left(a\right)}{\leq }R_{f}^{\prime }\left(d\right)\overset{\left(b\right)}{=}{\tilde{R}}^{\prime }\left(f\left(d\right)\right)\overset{\left(c\right)}{=}\tilde{R}\left(f\left(d\right)\right) \end{array}$$

where (a) follows from assumption (2) and Theorem A1 in the Appendix A, (b) is shown in Lemma 1, and (c) is due to [14] (see also ([13], Theorem 5.9.1)). The other direction,

(38) $$\begin{array}{c} R_{f}\left(d\right)\geq \tilde{R}\left(f\left(d\right)\right) \end{array}$$

is a consequence of the strong converse by Kieffer [12], see Lemma A1 in the Appendix A. ☐

An immediate application of Theorem 1 gives the f-separable rate-distortion function for several well-known binary memoryless sources (BMS).

**Example** **1** (BMS, Hamming distortion). *Let X be the binary memoryless source. That is, $X=\hat{X}=\left\{0,1\right\}$, $X_{i}$ is a Bernoulli(p) random variable, and d(·,·) is the usual Hamming distortion measure. Then, for any continuous increasing f(·) and $p\leq \frac{1}{2}$,*

$$R_{f}\left(d\right)=\left\{\begin{array}{cc} h\left(p\right)-h\left(\frac{f(d)-f(0)}{f(1)-f(0)}\right), & \frac{f(d)-f(0)}{f(1)-f(0)}<p\\ 0, & o.w. \end{array}\right\}$$

*where*

(39) $$\begin{array}{c} h\left(p\right)=p\log\frac{1}{p}+\left(1-p\right)\log\frac{1}{1-p} \end{array}$$

*is the binary entropy function. The result follows from a series of obvious equalities,*

(40) $$\begin{array}{cc} R_{f}\left(d\right) & =\underset{P_{\hat{X}|X}:E\left[f\left(d(X,\hat{X})\right)\right]\leq f\left(d\right)}{\inf}I\left(X;\hat{X}\right) \end{array}$$

(41) $$\begin{array}{c} =\underset{P_{\hat{X}|X}:\frac{E\left[f\left(d(X,\hat{X})\right)\right]-f\left(0\right)}{f(1)-f(0)}\leq \frac{f(d)-f(0)}{f(1)-f(0)}}{\inf}I\left(X;\hat{X}\right) \end{array}$$

(42) $$\begin{array}{c} =\underset{P_{\hat{X}|X}:E\left[\frac{f\left(d(X,\hat{X})\right)-f\left(0\right)}{f(1)-f(0)}\right]\leq \frac{f(d)-f(0)}{f(1)-f(0)}}{\inf}I\left(X;\hat{X}\right) \end{array}$$

(43) $$\begin{array}{c} =\underset{P_{\hat{X}|X}:E\left[d(X,\hat{X})\right]\leq \frac{f(d)-f(0)}{f(1)-f(0)}}{\inf}I\left(X;\hat{X}\right) \end{array}$$

(44) $$\begin{array}{c} =R\left(\frac{f(d)-f(0)}{f(1)-f(0)}\right). \end{array}$$

**Figure 2:**
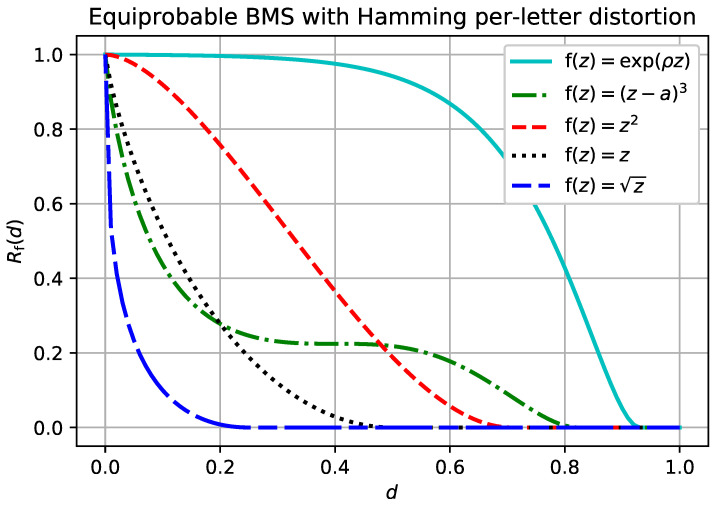
$R_{f}\left(d\right)$ for the binary memoryless source with p=0.5. Compare these to the f-separable distortion measures plotted for the binary source with Hamming distortion in Figure 1.

The rate-distortion function given in Example 1 is plotted in Figure 2 for different functions f. The simple derivation in Example 1 could be applied to any source for which the single-letter distortion measure can take on only two values, as is shown in the next example.

**Example** **2** (BMS, Erasure distortion). *Let X be the binary memoryless source and let the reconstruction alphabet have the erasure option. That is, X={0,1}, $\hat{X}=\left\{0,1,e\right\}$, and $X_{i}$ is a Bernoulli$\left(\frac{1}{2}\right)$ random variable. Let d(·,·) be the usual erasure distortion measure:*

$$d\left(x,\hat{x}\right)=\left\{\begin{array}{cc} 0, & x=\hat{x}\\ 1, & \hat{x}=e\\ \infty , & o.w. \end{array}\right\}.$$

*The separable rate-distortion function for the erasure distortion is given by*

R(d)=1−d,

*see ([11], Problem 10.7). Then, for any continuous increasing f(·),*

$$R_{f}\left(d\right)=1-\frac{f(d)-f(0)}{f(1)-f(0)}.$$

**Figure 3:**
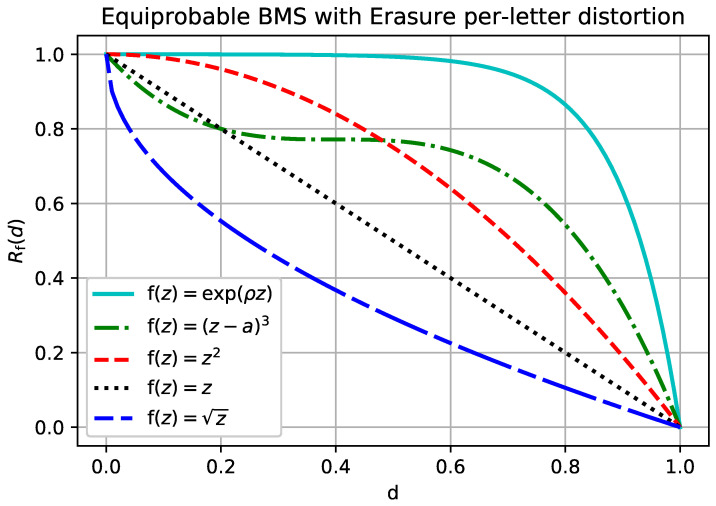
$R_{f}\left(d\right)$ for the binary memoryless source with p=0.5 and erasure per-letter distortion.

The rate-distortion function given in Example 2 is plotted in Figure 3 for different functions f. Observe that for concave f (i.e., subadditive distortion) the resulting rate-distortion function is convex, which is consistent with [10]. However, for f that are not concave, the rate-distortion function is not always convex. Unlike in the conventional separable distortion measure, an f-separable distortion measure is not convex in general.

**Figure 4:**
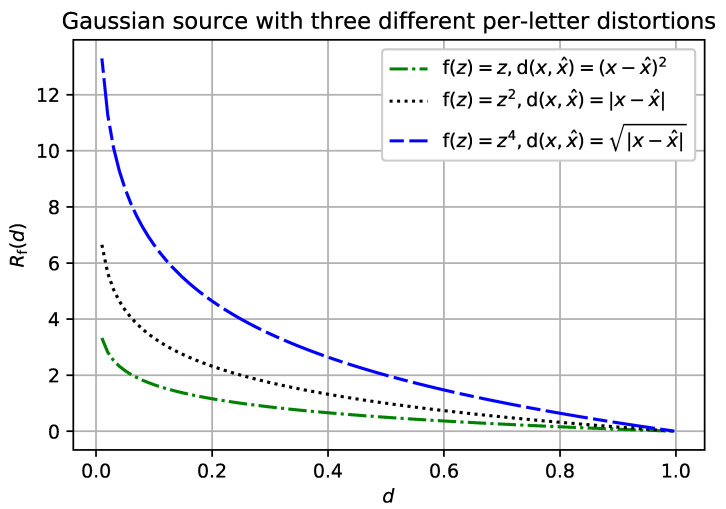
$R_{f}\left(d\right)$ for the Gaussian memoryless source with mean zero and unit variance.

Having a closed-form analytic expression for a separable distortion measure does not always mean that we could easily derive such an expression for an f-separable distortion measure with the same per-letter distortion. For example, consider the Gaussian source with the mean-square-error (MSE) per-letter distortion. According to Theorem 1, letting $f\left(z\right)=\sqrt{z}$ recovers the Gaussian source with the absolute value per-letter distortion. This setting, and variations on it, is a difficult problem in general [15]. However, we can recover the f-separable rate-distortion function whenever the per-letter distortion d(·,·) composed with f(·) reconstructs the MSE distortion, see Figure 4.

Theorem 1 shows that for well-behaved stationary ergodic sources, $R_{f}\left(d\right)$ admits a simple characterization. According to Lemma 1, the same characterization holds for the excess distortion paradigm without stationary and ergodic assumptions. The next example shows that, in general, $R_{f}\left(d\right)\neq \tilde{R}\left(f\left(d\right)\right)$ within the average distortion paradigm. Thus, assumption (1) is necessary for Theorem 1 to hold.

**Example** **3** (Mixed Source). *Fix λ∈(0,1) and let the source X be a mixture of two i.i.d. sources,*

(45) $$\begin{array}{c} P_{X^{n}}\left(x^{n}\right)=\lambda \prod_{i=1}^{n}P^{1}\left(x_{i}\right)+\left(1-\lambda \right)\prod_{i=1}^{n}P^{2}\left(x_{i}\right). \end{array}$$

*We can alternatively express X as*

(46) $$\begin{array}{c} X^{n}=ZX_{1}^{n}+\left(1-Z\right)X_{2}^{n} \end{array}$$

*where Z is a Bernoulli(λ) random variable. Then, the rate-distortion function for the mixture source (45) and continuous increasing f is given in Lemma A2 in the Appendix B. Namely,*

(47) $$\begin{array}{c} R_{f}\left(d\right)=\min_{\left(d_{1},d_{2}\right):\lambda d_{1}+\left(1-\lambda \right)d_{2}\leq d}\max\left\{R_{f}^{1}\left(d_{1}\right),R_{f}^{2}\left(d_{2}\right)\right\} \end{array}$$

*where $R_{f}^{1}\left(d\right)$ and $R_{f}^{2}\left(d\right)$ are the rate-distortion functions for discrete memoryless soruces given by $P^{1}$ and $P^{2}$, respectively. Likewise,*

(48) $$\begin{array}{c} \tilde{R}\left(f(d)\right)=\min_{\left(d_{1},d_{2}\right):\lambda d_{1}+\left(1-\lambda \right)d_{2}\leq f\left(d\right)}\max\left\{{\tilde{R}}^{1}\left(d_{1}\right),{\tilde{R}}^{2}\left(d_{2}\right)\right\}. \end{array}$$

**Figure 5:**
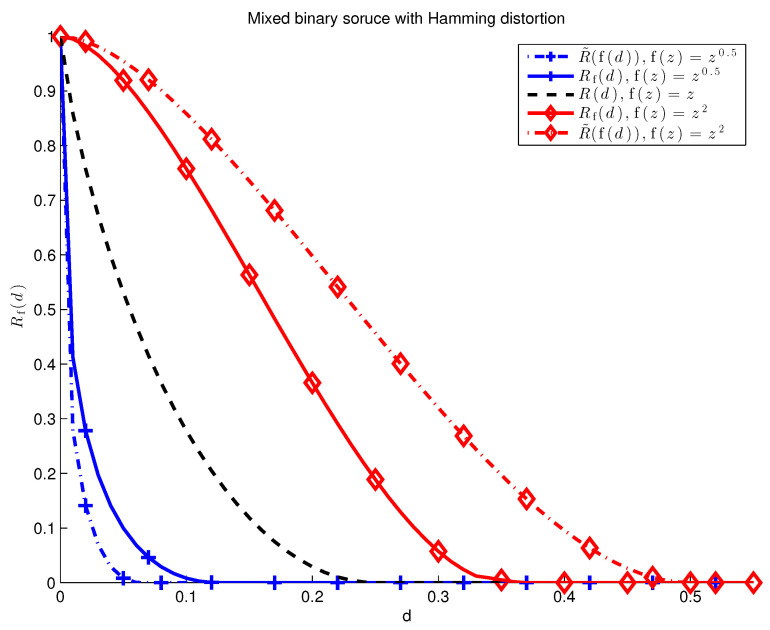
Mixed binary source with $p_{1}=0.5$, $p_{2}=0.001$, and λ=0.5. Three examples of f-separable rate-distortion functions are given. For f(z)=z, the relation $R\left(d\right)=\tilde{R}\left(d\right)$ follows immediately. When f is not the identity, $R_{f}\left(d\right)\neq \tilde{R}\left(f\left(d\right)\right)$ in general for non-ergodic sources.

As shown in Figure 5, Equations (47) and (48) are not equal in general.

## 4. Discussion

### 4.1. Sub-Additive Distortion Measures

Recall that an f-separable distortion measure is sub-additive if f is concave (cf. Section 1.3). Clearly, not all f-separable distortion measures are sub-additive, and not all sub-additive distortion measures are f-separable. An examplar of a sub-additive distortion measure (which is not f-separable) given in ([10], Chapter 5.2) is

(49) $$\begin{array}{c} d^{n}\left(x^{n},{\hat{x}}^{n}\right)=\frac{1}{n}{\left(\sum_{i=1}^{n}d^{q}\left(x_{i},{\hat{x}}_{i}\right)\right)}^{1/q},\;q>1. \end{array}$$

The sub-additivity of (49) follows from the Minkowski inequality. Comparing (49) to a sub-additive, f-separable distortion measure given by

(50) $$\begin{array}{c} d^{n}\left(x^{n},{\hat{x}}^{n}\right)={\left(\frac{1}{n}\sum_{i=1}^{n}d^{q}\left(x_{i},{\hat{x}}_{i}\right)\right)}^{1/q},\;0\leq q\leq 1, \end{array}$$

we see that the discrepancy between (49) and (50) has to do not only with the different ranges of *q* but with the scaling factor as a function of *n*.

Consider a binary source with Hamming distortion and let $x^{n}=0^{n},{\hat{x}}^{n}=1^{n}$. Rewriting (49) we obtain

(51) $$\begin{array}{c} d^{n}\left(x^{n},{\hat{x}}^{n}\right)=\frac{1}{n^{(q-1)/q}}{\left(\frac{1}{n}\sum_{i=1}^{n}d^{q}\left(x_{i},{\hat{x}}_{i}\right)\right)}^{1/q} \end{array}$$

and

(52) $$\begin{array}{cc} \lim_{n\to \infty }d^{n}\left(x^{n},{\hat{x}}^{n}\right) & =\lim_{n\to \infty }\frac{1}{n^{(q-1)/q}}{\left(\frac{1}{n}\sum_{i=1}^{n}d^{q}\left(0,1\right)\right)}^{1/q} \end{array}$$

(53) $$\begin{array}{c} =\lim_{n\to \infty }\frac{1}{n^{(q-1)/q}}{\left(\frac{1}{n}\sum_{i=1}^{n}1\right)}^{1/q} \end{array}$$

(54) $$\begin{array}{c} =\lim_{n\to \infty }\frac{1}{n^{(q-1)/q}}=0. \end{array}$$

In the binary example, the limiting distortion of (49) is zero even when the reconstruction of $x^{n}$ gets every single symbol wrong. It is easy to observe that example (49) is similarly degenerate in many cases of interest. The distortion measure given by (50), on the other hand, is an example of a non-trivial sub-additive distortion measure, as can be seen in Figure 2 and Figure 3 for $q=\frac{1}{2}$.

### 4.2. A Consequence of Theorem 1

In light of the discussion in Section 1.1, an alert reader may consider modifying (16) to

(55) $$\begin{array}{c} f^{-1}\left(E\left[f\left(d(X,c(g(X))\right)\right]\right)\leq d, \end{array}$$

and studying the (M,d)-lossy source codes under this new paradigm. Call the corresponding rate-distortion function $R^{f}\left(d\right)$ and assume that *n*-letter distortion measures are separable. Thus, at block length *n* the constraint (55) is

(56) $$\begin{array}{c} E\left[f\left(\frac{1}{n}\sum_{i=1}^{n}d\left(X_{i},{\hat{X}}_{i}\right)\right)\right]\leq f\left(d\right) \end{array}$$

where $\hat{X}=c\left(g\left(X\right)\right)$. This is equivalent to the following constraints:

(57) $$\begin{array}{cc} E\left[f\left(\frac{1}{n}\sum_{i=1}^{n}f^{-1}\left(\tilde{d}\left(X_{i},{\hat{X}}_{i}\right)\right)\right)\right] & \leq f(d) \end{array}$$

(58) $$\begin{array}{cc} \mathrm{and}\;E\left[{\tilde{d}}^{n}\left(X_{i},{\hat{X}}_{i}\right))\right] & \leq f(d) \end{array}$$

where ${\tilde{d}}^{n}$ is an $f^{-1}$-separable distortion measure. Putting these observations together with Theorem 1 yields

(59) $$\begin{array}{c} R^{f}\left(d\right)={\tilde{R}}_{f^{-1}}\left(f\left(d\right)\right)=R\left(f^{-1}\left(f\left(d\right)\right)\right)=R\left(d\right). \end{array}$$

A consequence of Theorem 1 is that the rate distortion function remains unchanged under this new paradigm.

## 5. Conclusions

This paper proposes f-separable distortion measures as a good model for non-linear distortion penalties. The rate-distortion function for f-separable distortion measures is characterized in terms of separable rate-distortion function with respect to a new single-letter distortion measure, f(d(·,·)). This characterization is straightforward for the excess distortion paradigm, as seen in Lemma 1. The proof is more involved for the average distortion paradigm, as seen in Theorem 1. An important implication of Theorem 1 is that many prominant results in rate-distortion literature (e.g., Blahut-Arimoto algorithm) can be leveraged to work for f-separable distortion measures.

Finally, we mention that a similar generalization is well-suited for channels with non-linear costs. That is, we say that $b^{n}$ is an f-separable cost function if it can be written as

(60) $$\begin{array}{c} b^{n}\left(x^{n}\right)=f^{-1}\left(\frac{1}{n}\sum_{i=1}^{n}f\left(b(x_{i})\right)\right). \end{array}$$

With this generalization we can state the following result which is out of the scope of this special issue.

**Theorem** **2** (Channels with cost). *The capacity of a stationary memoryless channel given by $P_{Y|X}$ and f-separable cost function based on single-letter function b(x) is*

(61) $$\begin{array}{c} C_{f}\left(\beta \right)=\underset{P_{X}:E\left[f\left(b(X)\right)\right]\leq f\left(\beta \right)}{\sup}I\left(X;Y\right). \end{array}$$

## Appendix A. Lemmas for Theorem 1

Theorem A1 can be distilled from several proofs in literature. We state it here, with proof, for completeness; it is given in its present form in [16]. The condition of Theorem A1 applies when the source satisfies assumptions (1)–(3) in Section 3. This is a consequence of the ergodic theorem and continuity of f.

**Theorem** **A1.** *Suppose that the source and distortion measure are such that for any γ>0 there exists $0<{▵}_{\gamma }<\infty$ and a sequence $b_{1},b_{2},\cdots$ such that*

(A1) $$\begin{array}{c} E\left[d^{n}\left(X^{n},b^{n}\right)1\left\{{▵}_{\gamma }<d^{n}\left(X^{n},b^{n}\right)\right\}\right]\leq_{n}\gamma . \end{array}$$

If a rate-distortion pair (R,d) is achievable under the excess distortion criterion, it is achievable under the average distortion criterion.

**Proof.** Choose γ>0. Suppose there is a code $\left(g^{n},c^{n}\right)$ with *M* codewords that achieves

(A2) $$\begin{array}{c} \lim_{n\to \infty }P\left[d^{n}\left(X^{n},c^{n}\left(g^{n}\left(X\right)\right)\right)>d+\gamma \right]=0. \end{array}$$

We construct a new code $\left({\hat{g}}^{n},{\hat{c}}^{n}\right)$ with M+1 codewords:

(A3) $$\begin{array}{c} {\hat{c}}^{n}\left(m\right)=\left\{\begin{array}{cc} b^{n}, & \mathrm{if}m=0\\ c^{n}\left(m\right), & \mathrm{if}m=1,\cdots ,M, \end{array}\right. \end{array}$$

(A4) $$\begin{array}{c} {\hat{g}}^{n}\left(x^{n}\right)=\left\{\begin{array}{cc} 0, & \mathrm{if}d^{n}\left(x^{n},c^{n}\left(g^{n}\left(x^{n}\right)\right)\right)>d^{n}\left(x^{n},b^{n}\right)\\ g^{n}\left(x^{n}\right), & \mathrm{if}d^{n}\left(x^{n},c^{n}\left(g^{n}\left(x^{n}\right)\right)\right)\leq d^{n}\left(x^{n},b^{n}\right). \end{array}\right. \end{array}$$

Then

(A5) $$\begin{array}{c} d^{n}\left(x^{n},{\hat{c}}^{n}\left({\hat{g}}^{n}\left(x^{n}\right)\right)\right)=\min\left\{d^{n}\left(x^{n},c^{n}\left(g^{n}\left(x^{n}\right)\right)\right),d^{n}\left(x^{n},b^{n}\right)\right\}. \end{array}$$

For brevity denote,

(A6) $$\begin{array}{cc} V_{n} & =d^{n}\left(X^{n},{\hat{c}}^{n}\left({\hat{g}}^{n}\left(X^{n}\right)\right)\right) \end{array}$$

(A7) $$\begin{array}{cc} W_{n} & =d^{n}\left(X^{n},c^{n}\left(g^{n}\left(X^{n}\right)\right)\right) \end{array}$$

(A8) $$\begin{array}{cc} Z_{n} & =d^{n}\left(X^{n},b^{n}\right). \end{array}$$

Then,

$$\begin{array}{c} E\left[V_{n}\right]\leq E\left[V_{n}1\left\{V_{n}\leq d+\gamma \right\}\right] \end{array}$$

(A9) $$\begin{array}{c} +E\left[V_{n}1\left\{d+\gamma <V_{n}\leq {▵}_{\gamma }\right\}\right]+E\left[V_{n}1\left\{{▵}_{\gamma }<V_{n}\right\}\right] \end{array}$$

(A10) $$\begin{array}{c} \leq d+\gamma +{▵}_{\gamma }P\left[1\{d+\gamma <V_{n}\}\right]+E\left[V_{n}1\left\{{▵}_{\gamma }<V_{n}\right\}\right] \end{array}$$

(A11) $$\begin{array}{c} \leq d+\gamma +{▵}_{\gamma }P\left[1\{d+\gamma <W_{n}\}\right]+E\left[Z_{n}1\left\{{▵}_{\gamma }<Z_{n}\right\}\right] \end{array}$$

(A12) $$\begin{array}{c} \leq_{n}d+2\gamma +E\left[Z_{n}1\left\{{▵}_{\gamma }<Z_{n}\right\}\right] \end{array}$$

(A13) $$\begin{array}{c} \leq_{n}d+3\gamma . \end{array}$$

☐

The following theorem is shown in ([12], Theorem 1).

**Theorem** **A2** (Kieffer). *Let X be an information source satisfying conditions (1)–(3) in Section 3, with f being the identity. Let $d^{n}$ be separable. Given an arbitrary sequence of $\left(n,M_{n},d,\epsilon_{n}\right)$-lossy source codes, if*

(A14) $$\begin{array}{c} \lim_{n\to \infty }\frac{1}{n}\log M_{n}<R\left(d\right) \end{array}$$

*then*

(A15) $$\begin{array}{c} \lim_{n\to \infty }\epsilon_{n}=1. \end{array}$$

An important implication of Theorem A2 for f-separable rate-distortion functions is given in the following lemma.

**Lemma** **A1.** *Let X be an information source satisfying conditions (1)–(3) in Section 3. Then,*

(A16) $$\begin{array}{c} R_{f}\left(d\right)\geq \tilde{R}\left(f\left(d\right)\right) \end{array}$$

**Proof.** If $\tilde{R}\left(f\left(d\right)\right)=0$, we are done. Suppose $\tilde{R}\left(f\left(d\right)\right)>0$. Assume there exists a sequence ${\left\{\left(g_{n},c_{n}\right)\right\}}_{n=1}^{\infty }$ of $\left(n,M_{n},d_{n}\right)$-lossy source codes (under f-separable distortion) with

(A17) $$\begin{array}{c} \underset{n\to \infty }{\lim\sup}\frac{1}{n}\log M_{n}<\tilde{R}\left(f\left(d\right)\right) \end{array}$$

and

(A18) $$\begin{array}{c} \underset{n\to \infty }{\lim\sup}\;d_{n}\leq d. \end{array}$$

Since $\tilde{R}\left(f\left(d\right)\right)$ is continuous and decreasing, there exists some γ>0 such that

(A19) $$\begin{array}{c} \lim_{n\to \infty }\frac{1}{n}\log M_{n}<\tilde{R}\left(f\left(d+\gamma \right)\right)<\tilde{R}\left(f\left(d\right)\right). \end{array}$$

For every *n*, the $\left(g_{n},c_{n}\right)$ lossy source code is also an $\left(n,M_{n},d+\gamma ,\epsilon_{n}\right)$-lossy source code for some $\epsilon_{n}\in \left[0,1\right]$ and f-separable $d^{n}$. It is also an $\left(n,M_{n},f\left(d+\gamma \right),\epsilon_{n}\right)$-lossy source code with respect to separable distortion ${\tilde{d}}^{n}\left(\cdot ,\cdot \right)$. We can therefore apply Theorem A2 to obtain

(A20) $$\begin{array}{c} \lim_{n\to \infty }\epsilon_{n}=1. \end{array}$$

Thus,

(A21) $$\begin{array}{cc} d_{n}\geq E\left[d^{n}\left(X^{n},c^{n}\left(g^{n}\left(X^{n}\right)\right)\right)\right] & \geq \epsilon_{n}\left(d+\gamma \right), \end{array}$$

(A22) $$\begin{array}{c} >d+\frac{\gamma }{2} \end{array}$$

where (A22) holds for all sufficiently large *n*. The result follows since we obtained a contradiction with (A18). ☐

## Appendix B. Rate-Distortion Function for a Mixed Source

**Lemma** **A2.** *The rate-distortion function with respect to f-separable distortion for the mixture source (45) is given by*

(A23) $$\begin{array}{c} R_{f}\left(d\right)=\min_{\left(d_{1},d_{2}\right):\lambda d_{1}+\left(1-\lambda \right)d_{2}\leq d}\max\left\{R_{f}^{1}\left(d_{1}\right),R_{f}^{2}\left(d_{2}\right)\right\} \end{array}$$

*where $R_{f}^{1}\left(d\right)$ and $R_{f}^{2}\left(d\right)$ are the rate-distortion functions with respect to f-separable distortion for stationary memoryless sources given by $P^{1}$ and $P^{2}$, respectively.*

**Proof.** Observe that,

(A24) $$\begin{array}{cc} M_{f}^{*}\left(d\right) & \geq \min_{(d_{1},d_{2})\in D}\max\left\{M_{f}^{1}\left(d_{1}\right),M_{f}^{2}\left(d_{2}\right)\right\} \end{array}$$

(A25) $$\begin{array}{cc} M_{f}^{*}\left(d\right) & \leq \min_{(d_{1},d_{2})\in D}\max\left\{2M_{f}^{1}\left(d_{1}\right),2M_{f}^{2}\left(d_{2}\right)\right\} \end{array}$$

where

(A26) $$\begin{array}{c} D=\left\{\left(d_{1},d_{2}\right):\lambda d_{1}+\left(1-\lambda \right)d_{2}\leq d\right\}, \end{array}$$

$M_{f}^{1}\left(d_{1}\right)$ and $M_{f}^{2}\left(d_{1}\right)$ are the non-asymptotic limits for $P^{1}$ and $P^{2}$, respectively. Indeed, the upper bound follows by designing optimal codes for $P_{1}$ and $P_{2}$ separately, and then combining them to give

(A27) $$\begin{array}{cc} M_{f}^{*}\left(d\right) & \leq \min_{(d_{1},d_{2})\in D}\left\{M_{f}^{1}\left(d_{1}\right)+M_{f}^{2}\left(d_{2}\right)\right\} \end{array}$$

(A28) $$\begin{array}{c} \leq \min_{(d_{1},d_{2})\in D}\max\left\{2M_{f}^{1}\left(d_{1}\right),2M_{f}^{2}\left(d_{2}\right)\right\}. \end{array}$$

The lower bound follows by the following argument. Fix an (M,d)-lossy source code (f-separable distortion), (g,c). Define

(A29) $$\begin{array}{c} d_{1}=E\left[d^{n}\left(X^{n},c\left(g\left(X^{n}\right)\right)\right)|Z=0\right], \end{array}$$

(A30) $$\begin{array}{c} d_{2}=E\left[d^{n}\left(X^{n},c\left(g\left(X^{n}\right)\right)\right)|Z=1\right]. \end{array}$$

Clearly, $(d_{1},d_{2})\in D$. It also follows that

(A31) $$\begin{array}{c} M\geq M_{f}^{1}\left(d_{1}\right) \end{array}$$

since (g,c) is an $\left(M,d_{1}\right)$-lossy source code (f-separable distortion) code for $X_{1}^{n}$. Likewise,

(A32) $$\begin{array}{c} M\geq M_{f}^{2}\left(d_{2}\right) \end{array}$$

which proves the lower bound. The result follows directly from (A25). ☐
